# Exploring the Correlation Between Influenza A Virus (H3N2) Infections and Neurological Manifestations: A Scoping Review

**DOI:** 10.7759/cureus.36936

**Published:** 2023-03-30

**Authors:** Mithun K Reddy, Jayashankar CA, Venkataramana Kandi, Pooja M Murthy, Ganaraja V Harikrishna, Snigdha Reddy, Manish GR, Koshy Sam, Sai Teja Challa

**Affiliations:** 1 Medicine, Vydehi Institute of Medical Sciences and Research Centre, Bangalore, IND; 2 Internal Medicine, Vydehi Institute of Medical Sciences and Research Centre, Bangalore, IND; 3 Clinical Microbiology, Prathima Institute of Medical Sciences, Karimnagar, IND; 4 Neurology, Vydehi Institute of Medical Sciences and Research Centre, Bangalore, IND; 5 General Medicine, Vydehi Institute of Medical Sciences and Research Centre, Bangalore, IND

**Keywords:** febrile convulsions, encephalitis, neurological manifestations, h3n2, influenza a virus

## Abstract

Influenza A virus (IAV), particularly the H3N2 variant, is known to cause respiratory manifestations, but it can also lead to neurological complications ranging from mild symptoms like headache and dizziness to severe conditions such as encephalitis and acute necrotizing encephalopathy (ANE). In this article, the correlation between the H3N2 variant of the IAV and neurological manifestations is discussed. Additionally, prompt recognition and treatment of influenza-associated neurological manifestations are highlighted to prevent infection-related long-term complications. This review briefly discusses various neurological complications linked to IAV infections, such as encephalitis, febrile convulsions, and acute disseminated encephalomyelitis, and the potential mechanisms involved in the development of neurological complications.

## Introduction and background

The influenza virus is a segmented ribonucleic acid (RNA) virus that belongs to the *Orthomyxovirus *family. The myxovirus group shows a predilection towards mucus and mucus-secreting respiratory epithelial cells. There are different types of influenza viruses depending on their host preferences, the intensity of infection, and their ability to undergo mutations. Influenza viruses, especially the Influenza A virus (IAV) and Influenza B virus (IBV), have surface projections, also called spike proteins, that are made up of hemagglutinin (HA) and neuraminidase (NA). IAVs have been known to be frequent causes of infection outbreaks, including incidences of the pandemic, as evidenced by the 1918 Spanish flu pandemic caused by the H1N1 variant. Over the next century, more than 11 IAV variants were discovered from different parts of the world. Moreover, IAVs appear to adapt to multiple hosts, including birds and animals, and have zoonotic potential. The IBV, Influenza C virus (ICV), and Influenza D virus (IDV) appear in two, six, and zero lineages/variants, respectively. Among the influenza viruses, IAV has a higher rate of mutability due to the antigenic shifts or major genomic changes that the virus undergoes to adapt to different hosts. However, the IBV, ICV, and IDVs can only undergo antigenic drifts or minor genetic alterations (Table 1) [1-21].

**Table 1:** Influenza types, origins, host preferences, and neurologic manifestations of influenza viruses H/HA: Hemagglutinin; N/NA: Neuraminidase; RNA: Ribonucleic acid

Virus type	Variant	Year of discovery (common name)	Country	Host preference	Neurological manifestations
Type-A (8 segmented RNA) (Highly mutagenic-antigenic shift)	H1N1	1918, 1976 (swine flu/Spanish flu), 1977 (Russian flu), 2009	Spain, Alberta, Canada	Bird, duck	Headache, numbness, paresthesia, drowsiness, Guillain-Barre syndrome (GBS), polyneuropathy, seizures, coma
	H2N2	1957 (Asian flu)	Singapore, HongKong	Birds and pigs	No data
	H3N2	1968	America	Human	Seizures, encephalopathy
	H3N2v	2011	America	Pigs	Encephalitis
	H3N8	1889	America, Europe, China	Horses	No data
	H5N1	1997 (avian/bird flu)	China	Geese, poultry	Neuroinflammation, neurodegeneration,
	H5N5	2008	China	Ducks, poultry	No data
	H5N6	2017	Greece, Netherlands. Asia	Duck, birds	Acute encephalitis
	H5N8	2010	China	Poultry	Seizures and encephalitis in animals (fox)
	H7N7	2003	Europe	Poultry	Neuroinflammation and cognitive deficits in animal experiments
	H7N9	2013 (avian flu)	China	Poultry, ducks	Cytopathic effects on brain cells in tissue culture experiments
	H7N3	2003 (bird flu)	Taiwan, British Columbia, North America	Migratory birds, poultry	Encephalitis in animal experiments
	H9N2	1966 (bird flu)	America, Turkey	Chicken, duck, pigeon	Neurovirulence in animal experiments
	H10N8	2012 (bird flu)	China	Ducks	No data
Type-B (8 segmented RNA) (Moderately mutagenic-antigenic drift)	Have HA and NA	1988	Yamagata, Japan	Humans	Convulsions, encephalopathy, and neurologic complications in children and adults
	Yamagata and Victoria				
Type-C (7 segmented RNA) (Low-level mutations-antigenic drift)	No HA, and NA, but have hemagglutinin-esterase fusion (HEF) glycoprotein Six lineages Aichi/1/81, Yamagata/26/81, Mississippi/80, Taylor/1233/47, Sao Paulo/378/82, and Kanagawa/1/76^(21)^	1947	America, Japan	Humans, pigs, cows	No data
Type-D (7 segmented RNA) (Low-level mutations-antigenic drift)	No HA, and NA, but have HEF glycoprotein	2011	America	Pigs, cattle	Neurovirulence in animal studies

Despite the fact that influenza viruses are generally associated with flu-like illnesses, wherein patients develop respiratory symptoms including sore throat/pharyngitis and a runny nose, patients may also suffer from generalized complaints like fever and myalgia. However, recent findings point to the capability of influenza viruses to affect the brain and result in neurological manifestations such as encephalitis, encephalopathy, and acute necrotizing encephalopathy (ANE) [22,23].

## Review

Epidemiology

The incidence and prevalence of IAV vary from year to year, but the virus is responsible for a significant proportion of seasonal influenza cases. In recent years, H3N2 has been responsible for several severe influenza outbreaks. Influenza A has a seasonal pattern, with most cases occurring during the winter months in temperate regions. In tropical regions, influenza can occur year-round. Risk factors for influenza infection include age (older adults and young children are at higher risk), immunocompromised status, pregnancy, and certain underlying medical conditions such as chronic lung disease, heart disease, and diabetes [24].

The Myanmar H3N2 virus-based case studies using genome sequencing have noted that clade 3c.2 was predominant, with the circulation of 3C.2a, 3C.2a1, and 3C.2a1b. Moreover, the results of this study revealed that the World Health Organization (WHO) vaccine strain was different from the strain that was circulating. This points to the fact that the vaccine requires amendment, and further increased surveillance is necessary to understand the circulating viral variants [25]. India has been reporting increased incidences of H3N2 since the beginning of 2023. Moreover, H3N2 infection-related deaths are also being documented, which should be considered a cause for concern [26].

Neurological manifestation of influenza A H3N2

Influenza A H3N2 subtype has been associated with a range of neurological manifestations, from mild symptoms such as headache and dizziness to severe conditions like ANE, encephalitis, Guillain-Barré syndrome (GBS), and acute disseminated encephalomyelitis (ADEM) [23]. In children, influenza-related hospitalizations and intensive care unit (ICU) admissions were mostly associated with neurological manifestations, with a prevalence of 18% of influenza-positive children in one study. Patients with neurological manifestations had a 10 times higher risk of hospitalization than those without, and influenza A (H3N2) was the predominant strain associated with neurological manifestations [5].

In adult cases, influenza A (H3N2) has been reported to cause encephalitis, seizures, and meningoencephalitis. One rare case of a 38-year-old woman who presented with encephalitis and convulsions associated with H3N2 influenza infection was described. Computed tomography (CT) of the brain in this patient revealed a bilateral hypodense area and the magnetic resonance imaging (MRI) showed distinctive hyperintense regions in the thalamus [27]. In another case, a 51-year-old man with influenza-associated encephalitis (IAE) presented with fever and an imbalance of the mental condition [28]. A 48-year-old man with cephalalgia and low-grade fever developed a confusional state and was diagnosed with viral encephalitis despite normal CT and cerebrospinal fluid (CSF) findings. Influenza A H3N2 was later detected in a nasopharyngeal swab, and the patient underwent emergency decompressive craniectomy. The patient had a poor clinical evolution and died 72 hours after ICU admission [29].

IAV variant H3N2 has also been linked to immune-mediated necrotizing myopathy (IMNM) in a 60-year-old Japanese woman. The patient developed subacute progressive muscle pain and weakness in her proximal extremities, myoglobinuria, elevated serum liver enzymes, and creatinine kinase. Muscle biopsy revealed positive human influenza A (H3N2) and anti-signal recognition particle (SRP)-associated IMNM. The patient was treated with oral systemic corticosteroids and immunoglobulins and ultimately recovered [30].

In children, IAV has been associated with febrile convulsions, with a higher incidence of febrile seizures and repeated seizures in the same episode than other infections. Influenza-like illness during influenza epidemics was significantly associated with hospital admissions for febrile convulsions [31,32].

Milano et al. reported three cases of influenza pneumonia with neurological complications, in which patients were managed with medication use, tracheotomy, and high-flow oxygen therapy. Patient one had diffuse delta waves and mild chronic leukoencephalopathy, patient two had sharp delta activity on the left-T region with bilateral spreading and severe cortical atrophy and moderate chronic leukoencephalopathy, and patient three had interictal delta waves on the right F-T regions [33].

In a separate case, a 16-year-old female with suspected IAV variant H1N1 infection presented with altered consciousness, seizures, and bilateral brain hyperintensities. Despite treatment with multiple medications, her condition deteriorated rapidly, and she ultimately passed away. Further viral studies revealed IAV variant H3N2 and a diagnosis of ANE was considered based on clinical features, neuroimaging results, and isolation of the virus from a throat swab [34].

Pathogenesis and mechanisms of neurological manifestations

The pathogenesis of these neurological manifestations is not fully understood but is believed to involve direct invasion of the virus into the central nervous system (CNS), immune-mediated mechanisms, cytokine storm, and other potential mechanisms. Direct invasion of the virus into the CNS is one of the proposed mechanisms of influenza-associated neurological manifestations. The influenza virus has been found to be capable of infecting neurons and glial cells, leading to CNS damage. The virus can enter the CNS through the olfactory bulbs, which are in direct contact with the nasal cavity. Once inside the CNS, the virus can cause neuronal damage and inflammation, leading to neurological symptoms.

Immune-mediated mechanisms have also been proposed as possible pathogenesis of influenza-associated neurological manifestations. In response to the virus, the immune system can release cytokines and chemokines, leading to inflammation and tissue damage. The resulting immune response can cause damage to the CNS, leading to neurological symptoms [35].

Cytokine storm, a phenomenon in which the immune system overreacts and releases large amounts of cytokines, has also been proposed as a possible mechanism of influenza-associated neurological manifestations. This cytokine release can lead to widespread inflammation, tissue damage, and neurological symptoms [36].

**Figure 1:**
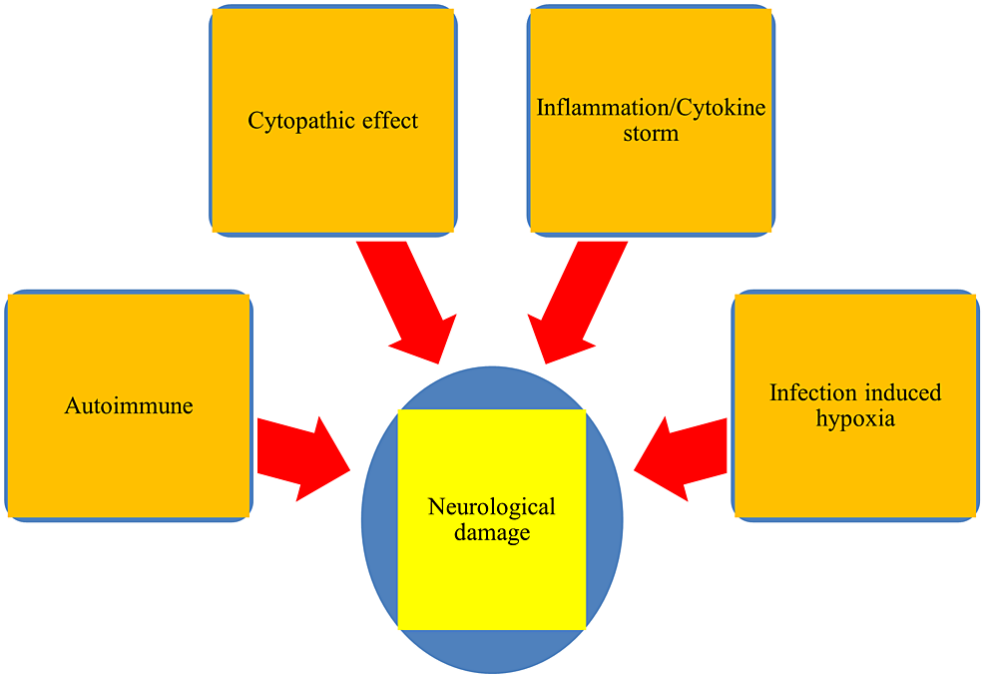
Mechanisms that potentially induce neurological damage following influenza virus infection

Other potential mechanisms of influenza-associated neurological manifestations include hypoxia, metabolic derangement, and vascular complications. Hypoxia, or oxygen deprivation, can occur as a result of respiratory failure caused by influenza, leading to CNS damage. Metabolic derangements, such as electrolyte imbalances and acidosis, can also occur in severe cases of influenza, leading to neurological symptoms. Vascular complications, such as stroke or thrombosis, can also occur as a result of influenza-associated inflammation [37]. The multiple mechanisms that can lead to neurological damage following IAV infections are depicted in Figure 1.

Diagnosis

In terms of diagnostic work-up and imaging modalities for influenza-associated neurological manifestations, clinical presentation and history are essential components. Patients with influenza-associated neurological symptoms should undergo a comprehensive neurological examination and a thorough evaluation of their medical history. Laboratory evaluation, such as CSF analysis, can also be useful in diagnosing influenza-associated neurological manifestations. Neuroimaging findings, such as MRI and/or CT scans, can also provide important information in diagnosing and managing these conditions [38].

Treatment and management

Treatment and management of influenza-associated neurological manifestations depend on the specific condition and its severity. General management strategies include supportive care, such as respiratory support and fluid management. Prevention strategies include vaccination against influenza, which can reduce the incidence and severity of influenza-associated neurological manifestations [39].

Antiviral medications against influenza viral infections which are currently available target the surface proteins including the NA and membrane channel protein (M2). The timely administration of antiviral drugs that target NA inhibitors, such as oseltamivir or zanamivir, can help to suppress viral replication and prevent the host inflammatory response from being further stimulated. This may potentially decrease the likelihood of influenza-related complications. It was observed that the drugs like amantadine and rimantadine that target the M2 surface proteins infiltrate sufficiently into the CSF. However, these drugs are infrequently prescribed due to their ability to develop primary resistance. Alternatively, therapeutic interventions utilizing immunomodulatory drugs and hypothermia-based therapies have been exploited experimentally [40].

## Conclusions

In conclusion, influenza viruses, particularly the A-type and its variants including the H3N2 subtype, are a predominant source of respiratory sickness globally. Available literature suggests a potential role of influenza A viral infection in the development of neurological symptoms. It's crucial to recognize the link between the currently circulating H3N2 IAV and neurological symptoms to recognize and manage these conditions appropriately. Early diagnosis and prompt management can enhance patient outcomes and avoid long-term complications. Given the significant morbidity and mortality associated with influenza-related neurological manifestations, it is critical for enhanced research in this direction.
